# The value of ultrasonographic factors in predicting cesarean following induction

**DOI:** 10.3389/fmed.2024.1430815

**Published:** 2024-10-31

**Authors:** Guangpu Liu, Chaofan Zhou, Zhifen Yang, Jingya Zhang

**Affiliations:** ^1^Department of Obstetrics, The Fourth Hospital of Hebei Medical University, Shijiazhuang, Hebei, China; ^2^Department of Neurology, Children’s Hospital of Hebei Province, Shijiazhuang, Hebei, China

**Keywords:** fetal head circumference, cesarean following induction, ultrasound, induction of labor, prediction model

## Abstract

This study aimed to develop and validate a prediction model of cesarean following induction of labor (IOL). A nomogram for the prediction of cesarean following IOL for singleton, cephalic term deliveries was created by comparing combinations of ultrasonographic and nonultrasonographic factors in a retrospective manner using patient data collected from a Chinese hospital between July, 2017 and December, 2023. Model discrimination and calibration were evaluated using the area under the receiver operating characteristic curve (AUROC) and a calibration curve. Subsequently, decision curve analysis (DCA) was conducted to pinpoint the optimal probability threshold for the predictive model to exhibit practical significance for clinical decision-making. A total of 738 women were included. The inclusion of ultrasound factors yielded a higher AUC when combined with nonultrasonographic factors. Of the three ultrasonographic factors analyzed, the most predictive factor for cesarean following IOL was fetal head circumference. After generating a nomogram with eight validated factors, including maternal age, gestational age, height, prior caesarean delivery, previous vaginal delivery, modified Bishop score, body mass index at delivery, and fetal head circumference by ultrasound, the trained and validated AUC values were 0.826 (95% confidence interval 0.786–0.867) and 0.883 (95% confidence interval 0.839–0.926), respectively. Decision curve analysis indicated that the model provided net benefits of between 0% and 80% of the probability threshold, indicating the benefits of using the model to make decisions concerning patients who fall within the identified range of the probability threshold. Our nomogram based on obstetric factors and fetal head circumference as obtained by ultrasound could be used to help counsel women who are considering IOL. The model demonstrates favorable net benefits within a probability threshold range of 0 to 80%.

## 1 Introduction

Induction of labor (IOL) is the artificial initiation of labor prior to its natural onset and stands as one of the most frequently conducted obstetrical procedures. IOL commonly carried out when the benefits of childbirth are deemed to outweigh the potential risks associated with prolonging the pregnancy. About 25% of all births necessitate the IOL (1), and this number is anticipated to rise in light of recently published studies that newly recommend IOL for various medical indications (2). Additionally, IOL is often considered for women over the age of 35, even in low-risk pregnancies, at 39 weeks’ gestation, as it has been shown that IOL does not lead to a higher rate of caesarean birth compared to expectant management (3, 4).

Unfortunately, exceeding 25% of IOLs fail to facilitate vaginal delivery and result in unintended cesarean sections (5–7) that both compromise the birth experience and heighten the likelihood of maternal and fetal complications, posing significant concerns. Therefore, it is important to predict the likely outcome of an IOL as accurately as possible on an individual basis in order to achieve the best maternal and infant outcomes.

To this end, multiple studies have developed models for predicting cesarean section after IOL (7–12), and most of them are based on obstetric factors measured during prenatal examinations or admission (7, 9–12). In addition, the use of single ultrasound data to predict cesarean section after induction is controversial as different studies have reported conflicting results (13–15). Hence, it appears viable to utilize maternal obstetric factors in conjunction with fetal ultrasound indicators in order to develop a more comprehensive prediction model. Currently, there has been limited research in China that combines obstetric factors with fetal ultrasound assessment (like fetal head circumference [HC], abdominal circumference [AC], as well as estimated fetal weight [EFW]) to predict the likelihood of cesarean section following IOL. Ultrasound measurements provide objective data, thus improving obstetricians’ ability to make informed clinical judgments during labor induction. The aim of our study was thus to establish a nomogram for the prediction of cesarean section among women who underwent IOL using the combinations of both ultrasonographic and non-ultrasonographic factors.

## 2 Materials and methods

We undertook a retrospective analysis of electronic medical records for pregnancies between July, 2017 and December, 2023 at a tertiary-level center in China. This study included women with singleton pregnancies in cephalic presentation who were induced after 36 completed weeks of gestation, irrespective of the indication for induction or cervical favorability. We excluded IOLs conducted for intrauterine fetal demise or major congenital malformations, those for women with more than one previous cesarean section, and those with missing ultrasound factor information.

Labor inductions were carried out similarly for all individuals. Although this was not controlled, standard management was used across the institution. The standard management technique was to begin with intracervical Foley balloon placement 12 h for cervical dilation. Then, 25 ug of misoprostol vaginally every 4 h at the providers’ discretion when additional cervical ripening was needed. Oxytocin was initiated once cervical ripening was completed or when > 4 misoprostol doses were used. Artificial rupture of membranes was performed at the discretion of the provider and Bishop scores were performed prior to cervical ripening.

The outcome for the prediction model was defined as cesarean birth for any indication. All the variables included in the model were derived from two previously published models (11, 12) on predictors of cesarean section following induction that have demonstrated the highest potential for application after prospective validation (16). Predictive variables include maternal age, gestational age, height, weight at birth; prior caesarean birth, previous vaginal birth, body mass index (BMI, body weight [kg]/height [m]^2^) at birth, as well as modified Bishop score (cervical dilation, station, and effacement). Considering the Han ethnic group represents most of the Chinese population, race was excluded a variable in this study.

The women included in the study had all undergone evaluation with ultrasonography for fetal biometry within one week prior to birth. Ultrasound scans were performed using a general electric voluson E6 machine (GE Healthcare, Zipf, Austria) with a con vex probe. Fetal biometry involved the assessment of fetal AC, fetal HC, and femur length (FL). Moreover, estimated fetal weight (EFW) was determined based on the Hadlock formula (17).

During the study period, comparisons were made between women who underwent IOL and had a cesarean delivery, and women who underwent IOL and had a vaginal birth, for all the aforementioned variables. Factors that showed statistical significance (*P* < 0.05, Supplementary Table 1) in the univariate comparisons were identified as potential candidates for inclusion in the predictive model.

Logistic regression was used to compare the odds ratios (ORs) of three ultrasound indicators within one week prior to the birth: fetal HC (mm), fetal AC (mm), and EFW (g). Due to collinearity, it was not possible to include all three indicators together in the logistic model. Consequently, we incorporated each ultrasound indicator individually into the model and identified the variable with the highest significant OR and area under the receiver operating characteristic (ROC) curve (AUC) as the optimal predictor. Next, we created a visual nomogram to depict the logistic regression model, utilizing the most significant OR value derived from the three ultrasound indicators in logistic regression analysis.

We estimated the required sample size for our predictive model following the simulations performed by Peduzzi et al. (18) and found that we needed to include at least 520 in the study. After we increased our initial calculation by 30% for validation purposes we reached a required sample size of 676 participants.

The model’s ability in discrimination and calibration were assessed through the AUC of the ROC as well as calibration curves. The result of final regression model was then visualized through an ROC curve. AUC together with asymptotic 95% confidence intervals (CI) were calculated to test the null hypothesis that the true area had a value of 0.5. Additionally, decision curve analysis (DCA) was performed to assess the net clinical benefit across various threshold probabilities of the predictive model with the highest discriminative capacity. SPSS 26.0 (IBM, Armonk, NY, USA) as well as STATA 17 (StataCorp College Station, TX, USA) were used to carry out all calculations.

Categorical variables were expressed as numbers and percentages, while continuous variables were expressed as mean ± standard deviation (SD). To assess the statistical significance of factors, Pearson’s chi-square test or Fisher’s exact test was used to analyze the differences in qualitative variables, and Student’s t test or the Kruskal-Wallis rank test was adopted to compare the differences in continuous variables.

The study was approved by the institutional review board of the Fourth Hospital of Hebei Medical University (2023KS280). Informed consent was waived since the electronic medical records alongside all information was processed anonymously. The data utilized did not contain any sensitive information such as the name, phone number, home address, or other identifying details of the individual patient. Finally, the study was conducted in accordance with the Declaration of Helsinki.

## 3 Results

**FIGURE 1:**
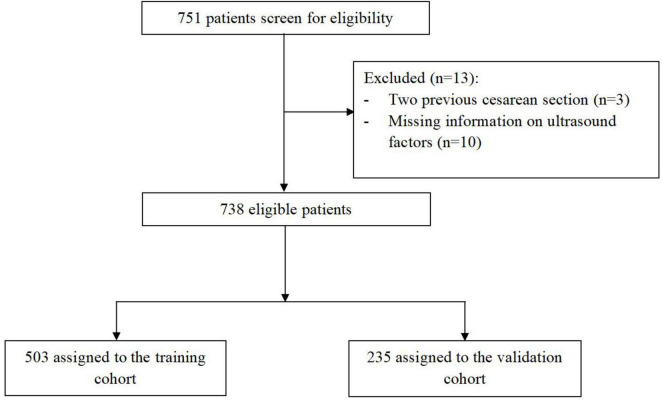
Flow of participants throughout the study.

A total of 738 women who underwent IOL at our hospital between July, 2017 and December 2023 were included in this analysis. Among them, 187 individuals (25.3%) underwent a cesarean section. The flow of participants through the study is depicted in Figure 1. Demographic and obstetric details can be found in Table 1 and Supplementary Table 1. Notably, no statistical variances (P > 0.05) were observed between the training and validation sets across all study variables, affirming the robustness of the grouping’s randomness.

**TABLE 1:** Demographic and obstetric characteristics of women undergoing IOL.

Demographic and obstetric characteristic	Training set (*n* = 503)^a^	Validation set (*n* = 235)^a^	*P*
Maternal age (year)	30.3 ± 3.7	30.5 ± 3.6	0.614
Gestational age (week)	39.3 ± 1.0	39.3 ± 1.1	0.717
Height (cm)	161.9 ± 4.5	162.1 ± 4.3	0.529
BMI at delivery (kg/m^2^)	28.7 ± 3.9	28.6 ± 3.9	0.739
Prior vaginal delivery			0.270
No	302(60.0)	131(55.7)	
Yes	201(40.0)	104(44.3)	
Prior caesarean delivery			0.891
No	491(97.6)	229(97.4)	
Yes	12(2.4)	6(2.6)	
Modified Bishop score	2.3 ± 1.4	2.3 ± 1.5	0.940
Estimated fetal weight (g)	3381.0 ± 338.7	3377.2 ± 326.7	0.886
Fetal head circumference (cm)	336.8 ± 16.5	337.4 ± 12.5	0.658
Fetal abdominal circumference (cm)	342.2 ± 16.9	341.6 ± 16.1	0.659

BMI, body mass index (calculated as weight in kilograms divided by the square of height in meters).

^a^Data are presented as mean ± standard deviation or as number (percentage).

Table 2 shows the predictive factors associated with cesarean after multivariate logistic regression of the frequency of cesarean in the training cohort on the three ultrasonographic factors together with nonultrasonographic factors. Model 1 included gestational age, maternal age, height, maternal BMI at birth, prior vaginal birth, prior caesarean birth, modified Bishop score, as well as fetal AC by ultrasound (AUC 0.822; 95% CI 0.780–0.863); Model 2 included gestational age, maternal age, height, maternal BMI at birth, prior vaginal birth, prior caesarean birth, modified Bishop score, as well as fetal HC by ultrasound (AUC 0.826; 95% CI 0.786–0.867); and Model 3 included gestational age, maternal age, height, maternal BMI at birth, prior vaginal birth, prior caesarean birth, modified Bishop score, as well as EFW by ultrasound (AUC 0.821; 95% CI 0.780– 0.863). A nonultrasonographic-factor-only model included maternal age, gestational age, height, maternal BMI at birth, prior vaginal birth, prior caesarean birth, as well as modified Bishop score (AUC 0.821; 95% CI 0.780–0.863) (Table 3). Notably, each ultrasound factor included (except EFW by ultrasound) reached a higher AUC when combined with nonultrasonographic factors, and Model 2 had a higher AUC in the training cohort.

**TABLE 2:** Predictors associated with cesarean following induction in multivariable logistic regression by three indicators of ultrasound in the training cohort (*n* = 503)^a^.

Variable	Non-ultrasonographic factors	*P*-value	Model 1^b^	*P*-value	Model 2^b^	*P*-value	Model 3^b^	*P*-value
Maternal age (year)	1.217 (1.126–1.314)	<0.001	1.217 (1.127–1.315)	<0.001	1.220 (1.129–1.319)	<0.001	1.217 (1.127–1.315)	<0.001
Gestational age (week)	1.419 (1.102–1.826)	0.007	1.448 (1.101–1.905)	0.008	1.311 (1.004–1.711)	0.047	1.474 (1.112–1.955)	0.007
Height (cm)	0.918 (0.869–0.970)	0.002	0.918 (0.869–0.970)	0.002	0.915 (0.866–0.968)	0.002	0.918 (0.869–0.971)	0.003
BMI at delivery (kg/m2)	1.111(1.046–1.180)	0.001	1.114(1.047–1.186)	0.001	1.101(1.036–1.170)	0.002	1.116(1.049–1.187)	< 0.001
Prior vaginal delivery	0.067 (0.033–0.134)	<0.001	0.066 (0.033–0.134)	<0.001	0.066 (0.033–0.134)	<0.001	0.066 (0.033–0.134)	<0.001
Prior caesarean delivery	5.504 (1.181–25.651)	0.030	5.629 (1.190–26.621)	0.029	5.561 (1.223–25.293)	0.026	5.539 (1.172–26.189)	0.031
Modified Bishop score	0.839 (0.712–0.989)	0.036	0.840 (0.713–0.990)	0.037	0.837 (0.709–0.987)	0.035	0.842 (0.715–0.993)	0.041
Indicators of fetal ultrasound	–		0.997 (0.982–1.013)	0.710	1.023 (1.001–1.045)	0.045	1.000 (0.999–1.001)	0.554

^a^Data are presented as odds ratio (95% confidence interval) unless otherwise stated.

^b^Model 1: fetal abdomen circumference by ultrasound (cm); Model 2: fetal head circumference by ultrasound (cm); Model 3: estimate fetal weight by ultrasound (g).

**TABLE 3:** Area and asymptotic 95% confidence interval under the ROC curve in the training cohort (*n* = 503).

Test result model^a^	Area	SE^b^	Asymptotic sig.^c^	Asymptotic 95%CI	
				Lower bound	Upper bound
Model 1	0.822	0.021	<0.001	0.780	0.863
Model 2	0.826	0.021	<0.001	0.786	0.867
Model 3	0.821	0.021	<0.001	0.780	0.863
Non-ultrasonographic factors	0.821	0.021	<0.001	0.780	0.863

CI, confidence interval; ROC, receiver operating characteristics; SE, standard error.

^a^Model 1: fetal abdomen circumference by ultrasound (cm); Model 2: fetal head circumference by ultrasound (cm); Model 3: estimate fetal weight by ultrasound (g).

^b^Under the nonparametric assumption.

^c^Null hypothesis: true area = 0.5.

**FIGURE 2:**
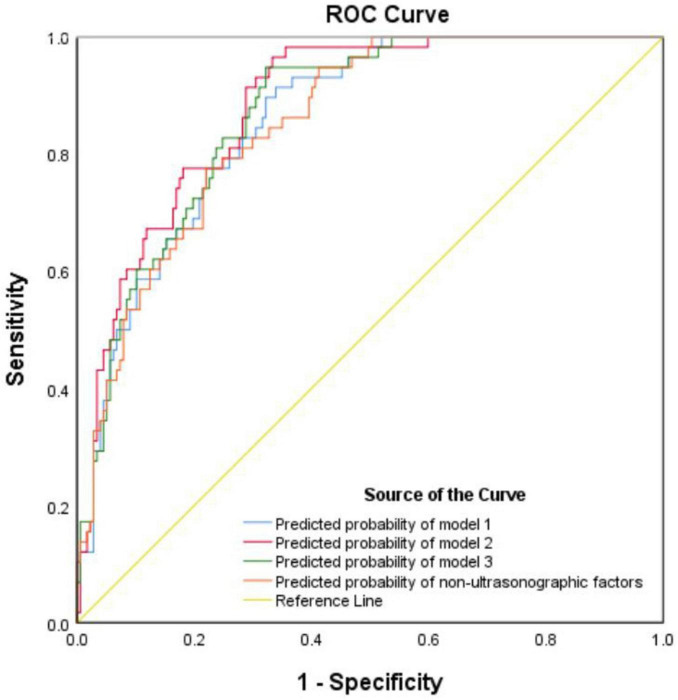
Receiver operating characteristic curve for prediction of cesarean following induction in the validation cohort.

**FIGURE 3:**
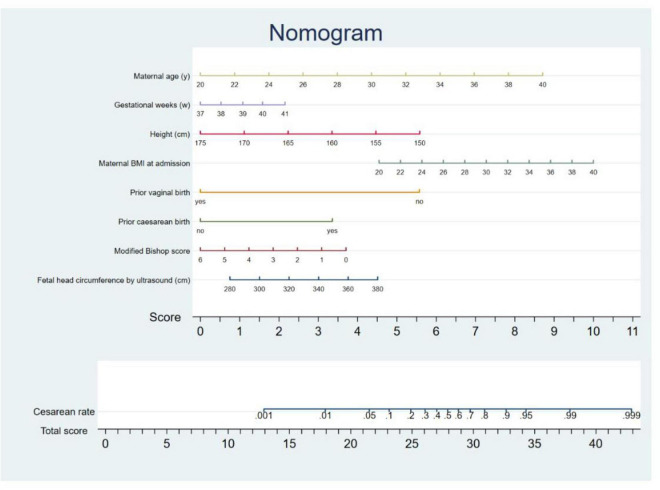
Nomogram for predicting the cesarean rate following induction.

Within the validation cohort, the AUC values achieved by models 1, 2, 3, and the model based on nonultrasonographic factors were 0.860, 0.883, 0.869, and 0.854, respectively (Table 4 and Figure 2). Notably, Model 2 had the largest AUC in the validation cohort. Consequently, we derived the predictive equation and constructed the nomogram based on the findings from Model 2. The equation derived from logistic regression to estimate the likelihood of cesarean delivery after IOL was as follows: predicted probability of cesarean following IOL = exp(w)/ [1 + exp(w)], where w is −12.741 + 0.199 × (maternal age) + 0.271 × (gestational weeks) −0.088 × (height) + 0.096 × (maternal BMI at the time of admission) −2.717 × (prior vaginal birth) + 1.716 × (prior cesarean birth) −0.179 × (modified Bishop score) + 0.022 × (fetal HC measured by ultrasound). On the basis of this established model, we subsequently developed a nomogram for visual reference (Figure 3).

**TABLE 4:** Area and asymptotic 95% confidence interval under the ROC curve in the validation cohort (*n* = 235).

Test result model^a^	Area	SE^b^	Asymptotic sig.^c^	Asymptotic sig.^c^	
				Lower bound	Upper bound
Model 1	0.860	0.025	<0.001	0.811	0.908
Model 2	0.883	0.022	<0.001	0.839	0.926
Model 3	0.869	0.024	<0.001	0.822	0.915
Non-ultrasonographic factors	0.854	0.025	<0.001	0.804	0.903

CI, confidence interval; ROC, receiver operating characteristics; SE, standard error.

^a^Model 1: fetal abdomen circumference by ultrasound (cm); Model 2: fetal head circumference by ultrasound (cm); Model 3: estimate fetal weight by ultrasound (g).

^b^Under the nonparametric assumption.

^c^Null hypothesis: true area = 0.5.

**FIGURE 4:**
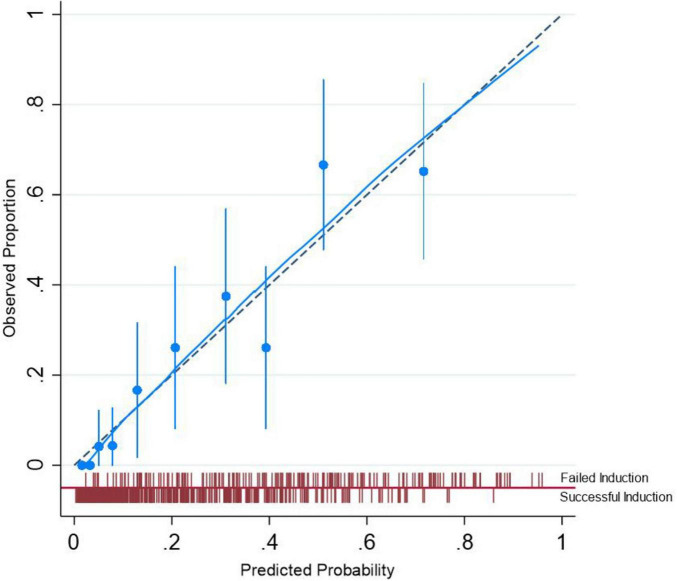
Calibration of the nomogram.

**FIGURE 5:**
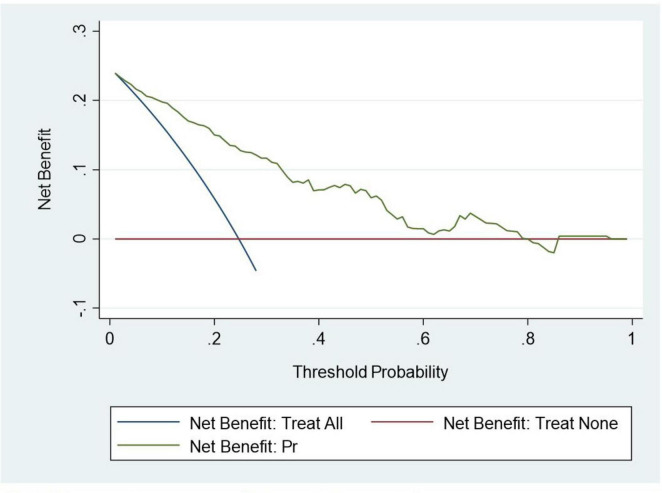
Decision curves of the predictive model.

The calibration plot for the validation cohort is presented in Figure 4. As illustrated, the predicted probability for cesarean following IOL was very close to the actual probability, and Hosmer-Lemeshow test results showed that this model was well calibrated (*P* = 0.28). Finally, DCA indicated that the model’s applicability was confined to threshold probabilities ranging from 0 to 0.8 (Figure 5).

## 4 Discussion

In this study we developed and validated a nomogram to predict the chance of cesarean for women undergoing IOL based on combinations of ultrasonographic and nonultrasonographic factors. We compared combinations of three ultrasound indicators (EFW, fetal HC, and fetal AC) with maternal factors, and found that the combination of fetal HC as measured by ultrasound with the maternal obstetric factors produced the most accurate predictions. The AUCs of this model were 0.826 (95% CI 0.786–0.867) and 0.883 (95% CI 0.839–0.926) for the training and validation cohorts, respectively.

Numerous predictive models of cesarean following IOL have already been reported for many countries, including China (7–12, 19, 20). Nevertheless, the majority of studies have been centered on maternal characteristics, with only a limited number incorporating EFW near term or fetal birth weight. To our knowledge, there have been currently no studies available that investigate the predictive capacity of fetal HC, fetal AC, or EFW derived from ultrasound imaging. Some scholars have established a prediction model for the cesarean rate following IOL, and most studies in this literature merely describe validations of these existing models with novel cohorts (8, 9, 11, 19). The Levine model (12), considered one of the most widely used in this context, has been validated in three different settings, with its AUC ranging from 0.61 to 0.76 in these studies (9, 16, 21). However, we believe that this and other models that were developed using Western populations are not directly applicable to Chinese populations due to notable differences in factors such as weight, height, fetal weight, pelvic size and IOL services between these demographic groups.

The model presented in this paper is a novel predictive model specifically validated on a Chinese cohort. This model integrates both non-ultrasonographic and ultrasonographic factors, allowing for personalized predictions tailored to individual women in this population. For pregnant women desiring for IOL, Ultrasound indicators can play a crucial role in boosting their confidence in IOL and can also aid obstetricians in making informed clinical decisions. Research has indicated that outcomes for both mothers and newborns following cesarean births after IOL are poorer compared to those of planned elective cesarean deliveries (22). Therefore, accurately identifying pregnant women with a low probability of cesarean following IOL is crucial for reducing maternal and neonatal complications. Our model has demonstrated applicability to the Chinese population and potentially to women with similar features in East Asia. Nevertheless, it is necessary to conduct further validation studies to ensure the model’s effectiveness and accuracy across other ethnicities.

Fetal size is a critical factor influencing the likelihood of successful vaginal birth following IOL in pregnant women with vertex singleton pregnancies. The estimation of fetal size encompasses factors such as estimated weight, AC, HC, and many other indicators. The application of ultrasound in the estimation of fetal size for the prediction of IOL remains controversial. D’Souza et al. (23) demonstrated that ultrasound-estimated third-trimester birth weights should not be included in a predictive model due to the fact that this measurement is not routinely determined in all pregnancies and because it has been shown to have a tendency to overestimate the true weight (24). Nevertheless, Jochum et al. (9), Danilack et al. (8), together with Migliorellia et al. (25) demonstrated that ultrasound estimation of macrosomia, excessive fetal growth, and ultrasound EFW were found to be independent factors related to the mode of birth after IOL, respectively.

There have been few studies on the effects of ultrasound measurement of fetal AC or HC on the outcome of induced labor. As routine ultrasound measurements taken prior to IOL in our clinical practice, this study showed that fetal HC, fetal AC, as well as EFW were all related to the mode of birth after IOL. However, due to the collinearity between different ultrasonic indicators, they cannot be placed together in the same prediction model. Therefore, it was only possible to compare these factors one-by-one and select the best predictor. After this one-by-one comparison, we proceeded to assess the predictive abilities of the three ultrasound parameters in conjunction with non-ultrasonographic maternal factors. Our analysis indicated that the optimal combination for prediction was fetal HC along with maternal factors.

In addition to ultrasound estimation of fetal size, there may be other ultrasound factors that can help predict cesarean birth after IOL. Pre-induction measurement of cervical length using transvaginal ultrasound is a well-known method for predicting the success of IOL, and some studies (26–29) have demonstrated that transvaginal ultrasonographic measurement of cervical length is a more accurate predictor of IOL success compared to the Bishop score. Additionally, a prospective observational study highlighted that the occiput posterior position, assessed via transabdominal ultrasound, was a significant independent predictor for cesarean birth with an OR of 5.7 and a p-value of 0.006 (30). Moreover, several parameters of transperineal ultrasound, a non-invasive imaging technique that provides a view of the pelvic anatomy through the perineum, have provided valuable insights into labor outcomes, including head-perineum distance, head-symphysis distance, as well as angle of progression (31–33). However, these ultrasonic indicators are not regularly checked or are only monitored after labor induction and were therefore not considered in this study.

The graphic nomogram in this paper was concise and easy to understand and could therefore be used to obtain the cesarean rate after IOL quickly. It was also convenient in clinical practice. The model predicted the probability of cesarean delivery for pregnant women undergoing IOL based on their demographic characteristics, obstetric background, and ultrasound data. Due to its high predictive accuracy, the model could offer objective and precise assistance to pregnant women and their obstetricians when selecting the birthing method. In particular, our findings indicated that the model yielded greater net benefits when the probability threshold ranged from 0% to 80%. This suggested that employing the model for decision-making regarding women within this probability range might be advantageous.

The main strength of our study is that we compared three fetal ultrasound indicators in order to obtain the most predictive combination of ultrasound and nonultrasound factors for cesarean after IOL. In addition, all variables in the nomogram can be easily measured prior to IOL. Importantly, the predictions from the model were most useful for women predicted to have less than an 80% chance of success. The study’s retrospective design posed a limitation, and its scope was restricted to the Han population in Shijiazhuang, Hebei Province. Furthermore, this study excluded individuals who underwent more than one previous cesarean section, as well as a lack of control over the administration of labor induction medications in the study, which may have had an impact on the results. Consequently, the findings may not be generalizable to the broader Chinese population. In addition, relationships between different characteristics and indications of cesarean birth should be further investigated in order to improve the nomogram if possible.

## 5 Conclusion

In conclusion, this study demonstrated that a combination of obstetric factors and fetal ultrasound indicators outperformed non-ultrasonographic factors in terms of AUC. Among the ultrasound indicators examined, fetal HC emerged as the most predictive factor for cesarean delivery following IOL. The nomogram developed using obstetric factors and fetal HC measured by ultrasound exhibited strong predictive capability in both the training (AUC = 0.826) and validation (AUC = 0.883) cohorts. This suggests its potential utility in providing guidance to women contemplating IOL. In addition, the predictions from the model proved most useful for patients predicted to have a < 80% chance of success. Nevertheless, we advise that additional external validation studies be undertaken with a substantial sample size across diverse populations before considering widespread clinical implementation of the model, so as to ensure the robustness and generalizability of the nomogram in varied clinical settings.

## Data Availability

The original contributions presented in the study are included in the article/Supplementary material, further inquiries can be directed to the corresponding author.

## References

[1] Rydahl E Eriksen L Juhl M. Effects of induction of labor prior to post-term in low-risk pregnancies: A systematic review. *JBI Database Syst Rev Implement Rep.* (2019) 17:170–208. 10.11124/JBISRIR-2017-003587 30299344 PMC6382053

[2] American College of Obstetricians and Gynecologists Committee on Obstetric Practice Society for Maternal-Fetal Medicine. Medically indicated late-preterm and early-term deliveries: ACOG committee opinion, number 831. *Obstet Gynecol.* (2021) 138:e35–9. 34259491 10.1097/AOG.0000000000004447

[3] Grobman W Rice M Reddy U Tita A Silver R Mallett G Labor induction versus expectant management in low-risk nulliparous women. *New Engl J Med.* (2018) 379:513–23. 30089070 10.1056/NEJMoa1800566 PMC6186292

[4] Walker K Bugg G Macpherson M McCormick C Grace N Wildsmith C Randomized trial of labor induction in women 35 years of age or older. *New Engl J Med.* (2016) 374:813–22. 26962902 10.1056/NEJMoa1509117

[5] Grobman W Bailit J Lai Y Reddy U Wapner R Varner M Defining failed induction of labor. *Am J Obstet Gynecol.* (2018) 218:122.e1-8. 10.1016/j.ajog.2017.11.556 PMC5819749 29138035

[6] Zenzmaier C Pfeifer B Leitner H König-Bachmann M. Cesarean delivery after non-medically indicated induction of labor: A population-based study using different definitions of expectant management. *Acta Obstet Gynecol Scand.* (2020) 100:220–8. 10.1111/aogs.13989 32880895

[7] Alavifard S Meier K Shulman Y Tomlinson G D’Souza R. Derivation and validation of a model predicting the likelihood of vaginal birth following labour induction. *BMC Pregnancy Childbirth.* (2019) 19:130. 10.1186/s12884-019-2232-8 30991983 PMC6469110

[8] Danilack V Hutcheon J Triche E Dore D Muri J Phipps M Development and validation of a risk prediction model for cesarean delivery after labor induction. *J Womens Health.* (2020) 29:656–69. 10.1089/jwh.2019.7822 PMC8935479 31657668

[9] Jochum F Le Ray C Blanc-Petitjean P Langer B Meyer N Severac F Externally validated score to predict cesarean delivery after labor induction with cervical ripening. *Obstet Gynecol.* (2019) 134:502–10. 31403585 10.1097/AOG.0000000000003405

[10] Overcash R Bauer D Auguste T Huang C Reddy U Kawakita T. Predicting vaginal delivery in nulliparous women undergoing induction of labor at term. *Am J Perinatol.* (2017) 35:660–8. 29212131 10.1055/s-0037-1608847

[11] Rossi R Requarth E Warshak C Dufendach K Hall E DeFranco E. Risk Calculator to Predict Cesarean Delivery Among Women Undergoing Induction of Labor. *Obstet Gynecol.* (2020) 135:559–68. 32028500 10.1097/AOG.0000000000003696

[12] Levine L Downes K Parry S Elovitz M Sammel M Srinivas SK. A validated calculator to estimate risk of cesarean after an induction of labor with an unfavorable cervix. *Am J Obstet Gynecol.* (2018) 218:254.e1-7. 10.1016/j.ajog.2017.11.603 PMC5807156 29224730

[13] Alanwar A Hussein S Allam H Hussein A Abdelazim I Abbas A Transvaginal sonographic measurement of cervical length versus Bishop score in labor induction at term for prediction of caesarean delivery. *J Matern Fetal Neonatal Med.* (2019) 34:2146–53. 31438737 10.1080/14767058.2019.1659770

[14] Tse W Chaemsaithong P Chan W Kwan A Huang J Appiah K Labor progress determined by ultrasound is different in women requiring cesarean delivery from those who experience a vaginal delivery following induction of labor. *Am J Obstet Gynecol.* (2019) 221:335.e1-18. 10.1016/j.ajog.2019.05.040 31153931

[15] De Miguel Manso S Colomo C Tejedor J Fontan J Real L Ramos L. Ultrasound examination of the cervix for predicting labor induction success: Failed validation in a routine clinical setting of a successful previous pilot study. *Arch Gynecol Obstet.* (2019) 301:75–84. 10.1007/s00404-019-05383-7 31745636

[16] López-Jiménez N García-Sánchez F Hernández-Pailos R Rodrigo-Álvaro V Pascual-Pedreño A Moreno-Cid M Risk of caesarean delivery in labour induction: A systematic review and external validation of predictive models. *BJOG Int J Obstet Gynaecol.* (2021) 129:685–95. 10.1111/1471-0528.16947 34559942

[17] Hadlock F Harrist R Carpenter R Deter R Park S. Sonographic estimation of fetal weight. The value of femur length in addition to head and abdomen measurements. *Radiology.* (1984) 150:535–40. 6691115 10.1148/radiology.150.2.6691115

[18] Peduzzi P Concato J Feinstein A Holford T. Importance of events per independent variable in proportional hazards regression analysis. II. Accuracy and precision of regression estimates. *J Clin Epidemiol.* (1995) 48:1503–10. 10.1016/0895-4356(95)00048-8 8543964

[19] Zhou H Gu N Yang Y Wang Z Hu Y Dai Y. Nomogram predicting cesarean delivery undergoing induction of labor among high-risk nulliparous women at term: A retrospective study. *BMC Pregnancy Childbirth.* (2022) 22:55. 10.1186/s12884-022-04386-8 35062898 PMC8783481

[20] Branger B Dochez V Gervier S Winer N. Césarienne après déclenchement du travail: Facteurs de risque et score de prédiction. *Gynécol Obstét Fertil Sénol.* (2018) 46:458–65. 29656953 10.1016/j.gofs.2018.03.008

[21] Alavifard S Meier K D’Souza R. Prediction calculator for induction of labor: No Holy Grail yet! *Am J Obstet Gynecol.* (2018) 219:419–20. 10.1016/j.ajog.2018.04.060 29752930

[22] Yang X Sun S. Comparison of maternal and fetal complications in elective and emergency cesarean section: A systematic review and meta-analysis. *Arch Gynecol Obstet.* (2017) 296:503–12. 28681107 10.1007/s00404-017-4445-2

[23] D’Souza R Ashraf R Foroutan F. Prediction models for determining the success of labour induction: A systematic review and critical analysis. *Best Pract Res Clin Obstet Gynaecol.* (2022) 79:42–54. 35090827 10.1016/j.bpobgyn.2021.12.005

[24] Milner J Arezina J. The accuracy of ultrasound estimation of fetal weight in comparison to birth weight: A systematic review. *Ultrasound.* (2018) 26:32–41. 29456580 10.1177/1742271X17732807 PMC5810856

[25] Migliorelli F Baños N Angeles Martina A Rueda C Salazar L Gratacós E Clinical and sonographic model to predict cesarean delivery after induction of labor at term. *Fetal Diagn Ther.* (2019) 46:88–96. 30293072 10.1159/000493343

[26] Rane S Guirgis R Higgins B Nicolaides K. The value of ultrasound in the prediction of successful induction of labor. *Ultrasound Obstet Gynecol.* (2004) 24:538–49. 15386612 10.1002/uog.1100

[27] Laencina A SÁNchez F Gimenez J MartÍNez M MartÍNez J VizcaÍNo V. Comparison of ultrasonographic cervical length and the Bishop score in predicting successful labor induction. *Acta Obstet Gynecol Scand.* (2010) 86:799–804. 10.1080/00016340701409858 17611824

[28] Park K Kim S Lee S Jeong E Jung H Oh K. Comparison between sonographic cervical length and Bishop score in preinduction cervical assessment: A randomized trial. *Ultrasound Obstet Gynecol.* (2011) 38:198–204. 10.1002/uog.9020 21484904

[29] Khalifa M Abbas A Gaber M Salah M. Bishop score versus transvaginal ultrasonographic measurement of cervical length in predicting successful labor induction in post-term pregnancy: Prospective cohort study. *Int J Reprod Contracept Obstet Gynecol.* (2018) 7:4646.

[30] Kamel R Negm S Youssef A Bianchini L Brunelli E Pilu G Predicting cesarean delivery for failure to progress as an outcome of labor induction in term singleton pregnancy. *Am J Obstet Gynecol.* (2021) 224:609.e1-11. 10.1016/j.ajog.2020.12.1212 33412128

[31] Hassan W Eggebø T Ferguson M Lees C. Simple two-dimensional ultrasound technique to assess intrapartum cervical dilatation: A pilot study. *Ultrasound Obstet Gynecol.* (2013) 41:413–8. 10.1002/uog.12316 23024020

[32] Hassan W Eggebø T Ferguson M Gillett A Studd J Pasupathy D The sonopartogram: A novel method for recording progress of labor by ultrasound. *Ultrasound Obstet Gynecol.* (2014) 43:189–94. 24105734 10.1002/uog.13212

[33] Youssef A Maroni E Ragusa A De Musso F Salsi G Iammarino M Fetal head–symphysis distance: A simple and reliable ultrasound index of fetal head station in labor. *Ultrasound Obstet Gynecol.* (2013) 41:419–24. 10.1002/uog.12335 23124698

