# Supplementary material for: The value of ultrasonographic factors in predicting cesarean following induction
Source: Front Med (Lausanne). 2024 Oct 31;11:1430815. doi: 10.3389/fmed.2024.1430815 (PMC11560775; doi:10.3389/fmed.2024.1430815)
Supplement: Supplementary file 1 [file Data_Sheet_1.docx]

**Table S. Demographic and obstetric characteristic of women undergoing IOL（n=738）**

| Demographic and Obstetric Characteristic | Vaginal birth  (n=551) | Cesarean delivery  (n=187) | P |
| --- | --- | --- | --- |
| Maternal age (year) | 30.2±3.7 | 30.8±3.6 | 0.034 |
| Gestational age (week) | 39.2±1.0 | 39.5±1.0 | 0.006 |
| Height (cm) | 162.5±4.2 | 160.5±4.7 | <0.001 |
| Weight at delivery (kg) | 74.8±10.8 | 76.6±10.7 | 0.054 |
| BMI at delivery (kg/m^2^) | 28.3±3.8 | 29.7±4.0 | <0.001 |
| Prior vaginal delivery |  |  | <0.001 |
| No | 270(49.0) | 163(87.2) |  |
| Yes | 281(51.0) | 24(12.8) |  |
| Prior caesarean delivery  No  Yes | 547(99.3)  4(0.7) | 173(92.5)  14(7.5) | < 0.001 |
| Modified Bishop score | 2.4±1.5 | 1.8±1.4 | < 0.001 |
| Estimated fetal weight (g) | 3364.6±321.8 | 3424.4±367.3 | 0.035 |
| Fetal head circumference (cm) | 335.8±16.4 | 340.4±11.3 | < 0.001 |
| Fetal abdominal circumference (cm) | 341.2±16.2 | 344.2±17.7 | 0.029 |

All unadjusted variables (*P*< 0.05) were entered into multivariable logistic models.
